# Alpha-1 antitrypsin is a potential biomarker for hepatitis B

**DOI:** 10.1186/1743-422X-8-274

**Published:** 2011-06-05

**Authors:** Xu-fei Tan, Shan-shan Wu, Shu-ping Li, Zhi Chen, Feng Chen

**Affiliations:** 1 State Key Laboratory of Infectious Disease Diagnosis and Treatment, First Affiliated Hospital, College of Medicine, Zhejiang University, Zhejiang, 310003 China; 2 Department of Pulmonary Diseases and Biomedical Research Center, Sir Run Run Shaw Hospital, College of Medicine, Zhejiang University, Zhejiang, 310003 China

## Abstract

**Background:**

Function exertion of specific proteins are key factors in disease progression, thus the systematical identification of those specific proteins is a prerequisite to understand various diseases. Though many proteins have been verified to impact on hepatitis, no systematical protein screening has been documented to hepatitis B virus (HBV) induced hepatitis, hindering the comprehensive understanding to this severe disease.

**Aim:**

To identify the major proteins in the progression of HBV infection from mild stage to severe stage.

**Methods:**

We performed an integrated strategy by combining two-dimensional electrophoresis (2-DE), peptide mass fingerprinting (PMF) analysis, and tissue microarray techniques to screen the functional proteins and detect the localization of those proteins.

**Results:**

Interestingly, MS/MS identification revealed the expression level of alpha-1 antitrypsin (AAT) was significantly elevated in serum samples from patients with severe chronic hepatitis. Immunoblotting with a specific AAT antibody confirmed that AAT is highly expressed in serum samples from patients with hepatic carcinoma and severe chronic hepatitis. Furthermore, we observed that AAT is with highest expression in normal tissue and cells, but lowest in hepatic carcinoma and severe chronic hepatitis tissues and cells, suggesting the specific secretion of AAT from tissues and cells to serum.

**Conclusion:**

These results suggest the possibility of AAT as a potential biomarker for hepatitis B in diagnosis.

## Introduction

Alpha-1 antitrypsin (AAT) is the most prominent protease inhibitor in human serum. More than 70 genetic variants of AAT have been described. It was documented that ATT deficiency associates with various types of liver diseases, such as neonatal hepatitis [1], cirrhosis and hepatoma [2].

Hepatitis B continues to be a worldwide clinical problem with approximately 300 million people chronically infected. Chronic infection is associated with significant morbidity and mortality as a result of long-term sequelae including inflammatory liver disease, cirrhosis, and hepatocellular carcinoma [3]. It's well known that different functions of specific proteins play crucial roles in hepatitis B virus (HBV) induced hepatitis, thus comprehensive identification of those specific proteins may greatly advance the disease diagnosis and biomarker search. To this end, many technologies were developed to identify disease associated proteins and biomarkers in diagnosis. Among them, proteomic analysis is a powerful tool to advance the diagnosis, treatment, and prevention of human diseases [4,5]. Two-dimensional electrophoresis (2-DE) is also widely used to identify biomarkers for diagnosis and therapeutic strategies. Steel *et al. *[6] constituted a proteomic approach for the discovery of early detection markers of hepatocellular carcinoma and Qing-Yu He *et al. *[7] used SELDI-ProteinChip combined with 2-DE to identify biomarkers in the serum samples of hepatitis B and hepatic carcinoma. However, these technology-dependent studies only displayed the expression of protein in the serum, but protein origin information was not provided, hindering the deep understanding to the investigated diseases.

In this study, we first applied 2-DE to screen specific proteins in the serum samples from mild and severe hepatitis B patients and alpha-1 antitrypsin (AAT) with high expression was identified. Using tissue microarray technique with few reagents [8], we confirmed the AAT identification by 2-DE. Furthermore, SELDI-TOF MC PMF was used to expand the protein information. Our studies thus provided useful information of the origin of AAT, which will be valuable for further explorations, especially specific amino acids.

## Materials and methods

### Patient Materials

Approved by the local ethics committee, 31 chronic hepatitis B patients (13 mild and 18 severe), 10 convalescent acute hepatitis B (AHB), 18 HBV-related Hepatocellular Carcinoma (HCC) patients and 12 healthy blood donors (normal controls) were enrolled in this study. The standards for diagnoses have been described previously [9]. All patients were HBsAg positive, and patients with hepatitis C, hepatitis D, human immunodeficiency virus type 1[HIV-1] positive and HIV-2 negative as well as with other chronic liver damages were excluded. Venous blood was collected and centrifuged at 2000 μg for 10 min. The supernatant were obtained and stored at -80°C.

### 2-DE protein separation

In order to identify the target proteins in the progress of HBV patients from mild to severe, one-milliliter of serum was collected from each patient of these two groups. The serum from mild and severe group were separately mixed. The serum albumin and IgG were removed using an albumin and IgG removal kit (GE healthcare, London, UK). The protein concentrations were determined by a Bradford assay. 2-DE was performed with IPGphor IEF (Amersham Biosciences, Uppsala, Sweden) and Ettan Dalt six electrophoresis units with the protocol suggested by the manufacturer. Isoelectric focusing (IEF) was performed using 240 mm IPG strips with IPGphor system. Two hundred micrograms of protein sample was diluted with rehydration solution (8 M urea, 2% (w/v) CHAPS, 0.5% (v/v) IPG buffer pH 4-7, 0.002% (w/v) bromophenol blue) to 450 μl and then loaded on the strip holder. Six gels were separated at once, three per group. The IPG gels were rehydrated for 12 hrs under 30 V at 20 W. IEF was performed with the following parameters: 500 V for 1 h, 1000 V for 1 hr, 8000 V for 8 hrs and 20 min. After IEF, the strips were equilibrated for 15 min in SDS equilibration buffer (6M urea, 2% (w/v) SDS, 50 mM Tris-HCl pH 8.8, 30% (v/v) glycerol, 0.002%(w/v) bromophenol blue) containing 100 mM DTT and then in SDS equilibration buffer containing 250 mM idoacetamide. After equilibration, the strips were loaded onto a vertical 12.5% (w/v) SDS-PAGE gels and sealed with 0.5% (w/v) agarose. The vertical electrophoresis was performed at 10 W with a condition of 5 W/gel for 30 min followed by 15 W/gel until the bromophenol blue dye reached the bottom of the gel.

### Silver staining and imaging of 2DE gels

The gels were fixed with 40% (v/v) ethanol and 10% (v/v) acetic acid over night, and then incubated in the sensitizing solution (30%(v/v) ethanol, 0.2%(w/v) sodium thiosulphate and 6.8% (w/v) sodium acetate) for 30 min. After washing three times with milli-Q water for 15 min each, the gels were stained with 0.25% (w/v) silver nitrate solution for 20 min. Development was performed in 2.5% (w/v) sodium carbonate with 0.04% (v/v) formaldehyde for 5 min. Stop solution (1.46%(w/v) EDTA) was used to terminate the reaction.

### In-gel trypsin digestion of proteins

The selected protein spots from all gels were excised and placed in Eppendorf tubes and digested as previously described [10]. After washing twice in milli-Q water, the gels were destained with a 1:1 mixture of 30 mM potassium ferricyanide and 100 mM sodium thiosulfate. The gels were then washed twice in milli-Q water, dehydrated with ACN and dried in a SpeedVac for 20 min. The gels were digested over night with 10 μl of 20 ng/μl sequencing grade trypsin at 37°C. The peptide fragments were extracted with 30 μl solution containing 50% (v/v) ACN and 5% (v/v) TFA. The solutions containing peptide fragments were dried in a lyophilizer and reconstituted by adding 10 μl of 0.1% (v/v) formic acid.

### MALDI-TOF-MS analysis of tryptic peptide

MS/MS analysis was performed using a Bruker-Daltonics AutoFlex TOF-TOF LIFT Mass Spectrometer (Bruker, Germany) operated in the delayed extraction and linear mode. The tryptic digest mixture was mixed with HCCA matrix. The MALDI spectra averaged over 50 laser shots. All mass spectra were calibrated externally by using a standard peptide mixture (HCCA). Internal calibration was performed using automatic digestion peaks of trypsin.

### Database searching and identification of proteins

Peptide mass fingerprints obtained by the MALDI-TOF MS were used to search NCBInr using Mascot software. The parameters used for the search were as follows: (1) peptide mass ranged from 1,000 to 3,000 U; (2) modifications were allowed for carbamidomethyl and oxidation; (3) one missed cleavage site was allowed; (4) mass accuracy was ± 1U, and (5) restriction was placed on the species of *Mus*. The criteria for positive identification of proteins were set as follows: (1) the MS match consisted of a minimum of four peptides; (2) the matched peptides covered at least 20% of the whole protein sequence, and (3) 100 ppm or better mass accuracy.

### Preparation of AAT polyclonal antibody

An anti-human AAT rabbit polyclonal antibody was generated by EPITOMICS (EPITOMICS, USA), recognizing the amino acid residues 301 to 314. The antibody generation procedure was described as previously [11].

### Western Blotting

Serum proteins (10 ug) from each patient of mild, severe CHB patients, acute hepatitis B patients, HCC patients, and healthy control groups were separated on 12% SDS-PAGE gels, and then transferred onto PVDF membranes. After incubating with blocking solution (TBS-T containing 5% non-fat milk) under room temperature for 2 hrs, the membranes were probed with anti-human AAT rabbit polyclonal antibody at 4°C overnight. After washing four times of 10 min each with TBS-T, anti-rabbit (horse-radish peroxidase) HRP secondary antibody was added for 1 hr at room temperature, and the antigen-antibody interaction was detected by ECL detection kit and exposed to X-ray film.

### Immunohistochemical analysis of tissue microarrays

A tissue microarray block with 24 tissue sample cores (each 1.5 mm in diameter) was purchased from Cybrdi (CC03-11-002, United States). The 24 tissue samples were formalin-fixed, paraffin-embedded liver samples consisting of six cases of multiple types of cancer with matched normal controls. Slides from the tissue microarray block were deparaffinized. For antigen retrieval, the sections were immersed in citrate buffer and processed in a scientific microwave oven at 95°C for 10 min. After pretreatment with biotin blocking, rabbit anti-human AAT rabbit polyclonal antibody was applied at a dilution of 1:1000 and incubated at 4°C overnight. Biotinylated goat anti-rabbit IgG (1:100 dilution; Dako, Gene Tech) was used as a secondary antibody. Negative controls were created by omitting the primary antibody.

Assessment of AAT was based on the percentage of positive stained cells on a 3-point scale (0, absence of staining; 1+, moderate staining; 2+, strong staining).

### Cell line culture, extraction, and western blotting

Chang cells, HepG2, Huh7, 7402, 7721 cells were cultured in DMEM medium supplemented with 10% (v/v) fetal bovine serum (FBS) and antibiotics. The cells were maintained in an incubator at 37°C with CO_2 _in humidified atmosphere. The cells at the exponential growth phase were harvested with trypsinization. Then cells were lysed with lysis buffer (30 mM Tris, pH7 .5, 150 mM sodium chloride, 1 mM phenylmethylsulfonyl fluoride, 1 mM sodium orthovanadate, 1% Nonidet P-40, 10% glycerol) [12] for 15 mins at 4°C, vortexed and centrifuged at 13000 rpm at 4°C for 30 min. The supernatants were mixed in Laemmli loading buffer, boiled for 10 min, and then subjected to SDS-PAGE gels.

## Results

### The screening of specific proteins associated with severe hepatitis B

**Figure 1:**
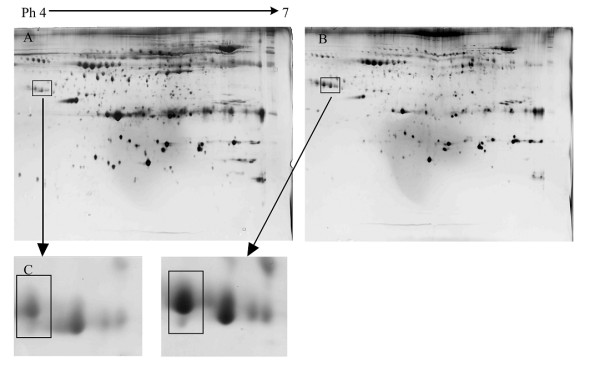
**2-DE images of serum proteins from patients with mild (A) and severe (B) chronic hepatitis**. The significantly higher expressed protein with 44kD (C) was further analysed by PMF.

To screen the proteins contributing to the progress of chronic hepatitis B (CHB), 2DE separation of serum protein was performed. 2-DE separation was performed three times for each sample of one group to minimize gel-to-gel variation (Figure 1A/B). Figure 1A showed the representative gel images from patients with mild CHB, while Figure 1B showed the image from patients with severe CHB. Spot size was compared between two gel images with ImageMaster. With the same quantity of total serum protein loading, it was clearly observed that the size of most spots in Figure 1B is smaller than those in Figure 1A except one area indicated in Figure 1C. Due to the unexpected observation, we next focused on those proteins in the following studies.

### Protein identification

**Figure 2:**
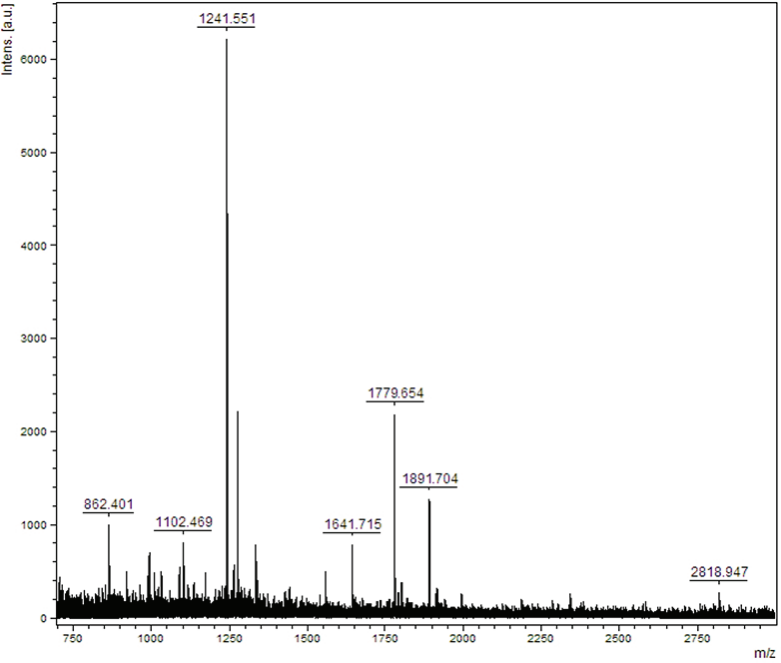
**MALDI-TOF MS spectrum of alpha-1 antitrypsin**. The major peaks are well matches to the protein sequence with probability based mowse score is 67. [Protein score is -10*Log(P), where P is the probability that the observed match is a random event. Protein scores greater than 65 are significant (p < 0.05).].

The spot highlighted in Figure 1C was excised and subjected to trypsin digestion followed by MALDI-TOF identification. Database searching suggested it was alpha-1 antitrypsin (AAT) (Table 1, Figure 2).

**Table 1:** MALDI-TOF identification and database search for protein identification

Protein	Peptides matched	Protein score	Experimental (MW)
alpha-1 antitrypsin	6	67	44179Da

### Western blot analysis of serum protein

**Figure 3:**
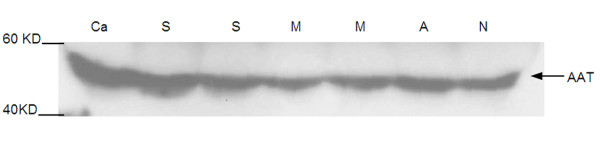
**The high expression of ATT in severe hepatitis B patients (Ca: hepatic carcinoma; S: severe hepatitis B; M: mild hepatitis B; A: acute hepatitis; N: normal)**.

To further confirm the protein identified by MALDI-TOF, western blotting was performed with the generated ATT antibody. In order to reveal the relationship between hepatitis B and AAT, more samples from different patients including healthy controls, acute hepatitis, and chronic hepatitis (including mild, severe, and hepatic carcinoma) were included. Among the serum samples from those groups, six samples in each group were randomly selected and subjected to the immunoblotting with anti-human AAT rabbit polyclonal antibody. With equal loading amount, highest ATT expression levels were in carcinama and severe chronic hepatitis B samples, but relatively lower in serum samples from normal and mild CHB patients (Figure 3), which is consitent with the results of 2-DE gel seperation.

### AAT immunostaining on tissue microarrays

**Figure 4:**
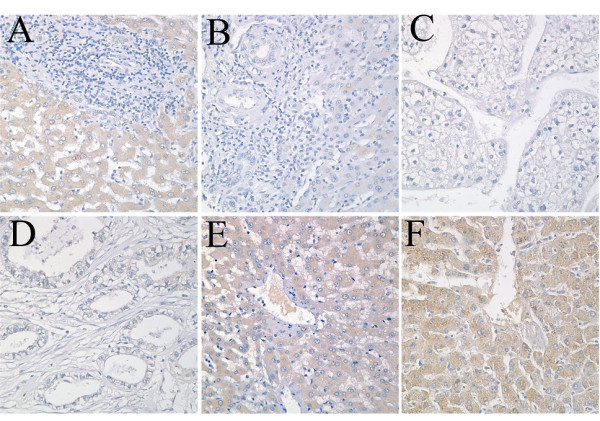
**Immunostaining for AAT on the tissue mircroarrays**. (A) Cholangiocarcinoma sample. (B) HCC sample. (C) Transparent HCC sample. (D) Moderate differentiated metastatic adenocarcinoma sample. (E) CHB sample. (F) Cancer adjacent normal liver tissue (set as positive control). (Original magnification: A to G, × 40).

Immunostaining for AAT was performed on the consecutive tissue microarray sliders. A total of 24 samples were analyzed, and 13 of those were divided into four types as cholangiocarcinoma, hepatocellular carcinoma, transparent hepatocellular carcinoma, and moderate differentiated metastatic adenocarcinoma. Eleven of those samples were noncancerous liver samples including chronic hepatitis and normal liver samples. Non-cancerous livers with chronic hepatitis and normal liver samples exhibit significant cytoplasmic staining of AAT compared with the HCC's (Figure 4). Even in the one slide of HCCs, the noncancerous area was found to be significantly expressed with AAT compared with the carcinoma areas.

### Western blotting analysis of protein from cell lines

**Figure 5:**
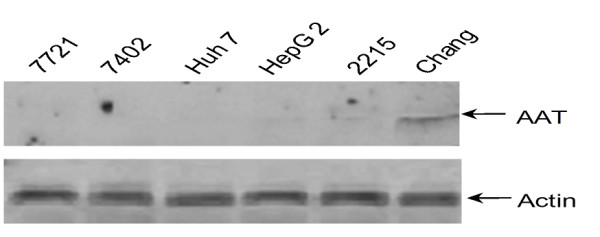
**AAT expression in different cell lines**. Six types of cells indicated were lysed and equal amount of proteins were subjected to SDS-PAGE. The fractionized proteins were blotted with anti-AAT and anti-Actin antibodies.

To further confirm the results from tissue microarrays, we next carried out western blotting in different cell lines including hepatic carcinoma cell line HepG2, 2215, HepG2, Huh7, BEL-7402 [13], SMMC-7721 [14] and normal hepatic cell line Chang [15]. With equal protein loading, proteins from all cell lines were subjected to the immunoblotting with anti-human AAT rabbit polyclonal antibody. As shown in Figure 5, the AAT signal was only detected in normal hepatic Chang cells, but not in other cell lines. These results are consistent with the data from tissue microarrays.

## Discussion

Identification of biomarkers with proteomic technology has become an important tool in biomedical research. The development of proteomics, especially two-dimensional electrophoresis associated with MS/MS, provides tremendous assistance to screen the biomarkers in various diseases. Many studies focus on biomarker identification for HBV infection [16,17], liver fibrosis, or cirrhosis that may result in HCC [18]. However, the methodology used in these studies only detect proteins in the serum, but not the tissues that secret or express these proteins.

J. Kenonen *et al. *[19] firstly introduced microarray technology in biomedical research, making hundreds of samples to be detected simultaneously by either immunohistochemistry or *in situ *hybridization on a single tissue slice [20,21]. As a result, in our study we combined 2-DE with high-throughput tissue microarray technology to study proteins that may be involved in the progression of mild hepatitis B infection to severe or even HCC in the patients. According to the result of SELDI-TOF MS PMS, we identified the highly expressed AAT in serum samples from patients with hepatic carcinoma severe hepatitis B.

To study the association between AAT and hepatitis B and HBV-related HCC, we prepared polyclonal antibody against AAT and performed western blotting exam. Results showed the gradual increase of serum AAT level from normal to hepatitis B patients and then to the HCC group. We further performed immunohistochemical analysis on hepatic tissue microarray slice. To verify the result of tissue chips, we further examined the AAT expression in different cell lines including HepG2 Huh7 7402 and 7721 hepatoma cell lines (Chang cells were set as normal control) by western blotting and the results showed AAT was only expressed in Chang cells but not other hepatitis cells or hepatic hepatoma cells. The discrepancy of ATT expression in serum and tissue/cell samples suggested the different mechanism by which ATT is expressed/secreted.

Blood plasma is with exceptional proteome in many respects, containing other tissue proteomes as subsets. As a widely used research subject, serum has its intrinsic advantages such as the convenience for preparation and rich aspects linking with various diseases. However, there are many disadvantages such as proteins in serum from various tissue proteomes, which makes serum not able to be used to determine the origins of disease associated proteins [22]. In our experiments, though the conclusion of AAT levels in serum, tissue and cell lines were not consistent, it is not difficult to understand that the high ATT level in serum may be secreted from hepatic carcinoma or hepatitis cells by certain inflammatory molecules or signal pathway activation, causing ATT levels in hepatic carcinoma or hepatitis cells invisible. On the other hand, the high level of AAT in serum may be derived from normal liver cells and represent self-protective responses of the liver cells as observed in other studies [23].

## List of abbreviations

AAT: alpha-1 antitrypsin; AHB: acute hepatitis B; CHB: chronic hepatitis B; HBV: hepatitis B virus; HCC: Hepatocellular Carcinoma; HRP: horse-radish peroxidase; IEF: Isoelectric focusing; PMF: peptide mass fingerprinting; 2-DE: two-dimensional electrophoresis

## Competing interests

No benefits in any form have been received or will be received from a commercial party related directly or indirectly to the subject of this article.

## Authors' contributions

CF and CZ proposed the study. TXF wrote the first draft and analyzed the data. All authors contributed to the design and interpretation of the study and to further drafts. CF is the guarantor. All authors have read and approved the final manuscript.
